# Potential of reproductive traits in functional ecology: A quantitative comparison of variability in floral, fruit, and leaf traits

**DOI:** 10.1002/ece3.11690

**Published:** 2024-07-18

**Authors:** Sonia Paź‐Dyderska, Andrzej M. Jagodziński

**Affiliations:** ^1^ Institute of Dendrology, Polish Academy of Sciences Kórnik Poland; ^2^ Poznań University of Life Sciences Faculty of Forestry and Wood Technology, Department of Game Management and Forest Protection Poznań Poland

**Keywords:** floral and fruit traits, organs, plant functional traits, reproduction, traits variability, trees and shrubs

## Abstract

Despite their claimed low intraspecific variability, plant reproductive traits are less frequently used in functional ecology. Here we focused on underrepresented plant organs, i.e. flowers and fruits, by comparing their traits with well‐established leaf traits. We evaluated 16 functional traits (six floral, six fruit, and four leaf traits) in a randomly selected group of woody species under comparable environmental conditions. We aimed to assess interspecific and intraspecimen variability and explore the potential of the proposed flower and fruit traits for ecological research. Traits related to the dry mass of flowers and fruits exhibited the highest interspecific variability, while carbon content traits in flowers and leaves had the lowest. At a specimen level, specific leaf area revealed the highest variation. Carbon content traits for all organs demonstrated the least intraspecimen variability, with flower carbon content being the least variable. Our study revealed connections between the newly proposed traits and widely recognized functional traits, uncovering intriguing links between the established traits and the floral and fruit traits upon which we focused. This complements the already well‐recognized variability in plant form and function with additional insights into reproductive processes.

## Graphical abstract

Reproductive traits, often overlooked in functional ecology, were the focus of our study. Comparing flower, fruit, and leaf traits in woody species, we discovered significant interspecific variability in flower and fruit traits alongside limited intraspecific variability. Our findings highlight the potential of these underrepresented reproductive traits to provide valuable insights into plant form and function, while also revealing connections with frequently used functional traits.

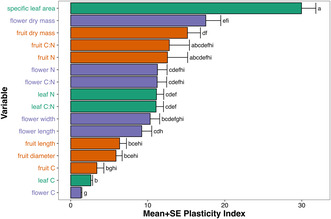

## 1 INTRODUCTION

Enhancing the predictive processes for future environmental changes is essential within the context of global climatic changes (Intergovernmental Panel on Climate Change, 2023). Improving these forecasts can be achieved through a more extensive and frequent integration of plant functional traits into ecological research (Liu et al., 2021). Plant functional traits encompass morphological, phenological, and physiological characteristics that define the growth, reproduction, and survival of plants, spanning from individual specimens, through populations and species, to entire plant communities (Díaz & Cabido, 2001; Lavorel & Garnier, 2002; Violle et al., 2007). The broad concept of functional traits offers significant utility for ecological studies, including the ability to predict ecosystem functioning and its responses to changing climates (Kühn et al., 2021; Liu et al., 2021), better understand species adaptations (Bussotti et al., 2015), assess biodiversity beyond species composition alone (Petchey & Gaston, 2006), inform conservation and restoration efforts (Ostertag et al., 2015), and assess resilience to disturbances and biological invasions (te Beest et al., 2015). Additionally, functional traits are valuable for the existing intercorrelations between them. Knowledge of the value of one trait often enables the assessment of another, even if measuring the latter involves greater difficulty, time, or effort (Díaz et al., 2016; Milla & Reich, 2007). Still, when considering trade‐offs between traits, it is essential to recognize that they result from different resource acquisition and allocation patterns, which are shaped by both community context and resource availability (Agrawal, 2020). Also, a holistic functional approach involving the traits of different plant organs can serve as a useful, quantified reflection of plant life strategies (Adler et al., 2014; Chai et al., 2016; Laughlin et al., 2010). Currently, many of the functional traits are used as tools for objective comparisons of different life strategies (Adler et al., 2014; Kattenborn et al., 2017) but also for predicting future changes in plant communities (Metcalfe et al., 2020; van Bodegom et al., 2014) or in their assembly (Laughlin, 2014; Laughlin et al., 2012).

Despite the numerous benefits derived from functional traits in ecological research, there are significant drawbacks related to the completeness and availability of functional data (Cordlandwehr et al., 2013; Johnson et al., 2021). The phylogenetic and geographic coverage of data about particular species is biased, predominantly favoring economically important woody species and regions in the Global North (Cornwell et al., 2019; Kattge et al., 2020). Additionally, even when a species is present in a database, it is often inadequately represented by a limited number of measurements, neglecting intraspecific variability (Siefert et al., 2015).

While functional research has extensively covered certain plant organs, such as leaves, stems, and seeds, there has been an uneven recognition of different plant organs (Paź‐Dyderska & Jagodziński, 2023). The leaf economic spectrum has guided further studies (Wright et al., 2004), preceded by the leaf‐height‐seed (LHS) concept by Westoby (1998). The study conducted by Díaz et al. (2016) effectively explained the variability of plant form and function at a global scale. However, recent studies emphasize the necessity for a deeper understanding of plant life strategies, urging a broader consideration of organs (Bardgett et al., 2014; Kleyer & Minden, 2015; Roddy et al., 2021), especially roots, which has prompted the dynamic development of this field of ecology, including the establishment of standardized protocols for root data collection and extensive root trait databases (Guerrero‐Ramírez et al., 2021; Iversen et al., 2017). Despite this progress, the coverage of organs through measurements remains uneven, with flowers and fruits being underrepresented and lacking standardized data collection protocols. So far, there have been limited papers on this topic, both for flowers (Bosch et al., 1997; Cresswell, 1998; Svensson, 1992) and fruits (Birkhold et al., 1992; Kourmpetli & Drea, 2014; Levey et al., 2000). Even fewer studies discuss flower physiology, anatomy, and morphology. Some of the questions still needing answers include potential threats to plant reproduction under changing climate conditions (Aun et al., 2024), the constraining role of water availability for flower maintenance (Lambrecht, 2013), and the vulnerability of flowers to xylem damage due to drought (Zhang & Brodribb, 2017). Major gaps in our knowledge of flowers and fruits may result from the lack of standardized data collection protocols, low species coverage, and unbalanced geographic and phylogenetic representation in data from different regions (E‐Vojtkó et al., 2020, 2022).

Recent years, however, have witnessed a positive shift, marked by an increasing number of studies focusing on floral (Chartier et al., 2017; Roddy, 2019; Roddy et al., 2021) and fruit traits (Acevedo‐Quintero et al., 2023; de Paz et al., 2018; Garrido et al., 2023). This trend is understandable, given that the diverse forms of flowers and fruits offer a wide range of valuable information. So far, several works discuss flower traits, which could significantly contribute to our understanding of plant biology and ecology. For example, flower traits such as reflectance, scent, and morphology can profoundly impact interactions with pollinators and herbivores (Junker & Parachnowitsch, 2015). Similarly, fruit traits can significantly affect ecological interactions. Factors such as size, color, and odor can influence interactions with dispersers, strongly impacting seed dispersal and colonization possibilities (Mahandran et al., 2023; Rodríguez et al., 2013). Furthermore, variation in floral and fruit traits can contribute to plant lineage diversification over evolutionary time as plants adapt to different ecological niches and selective pressures (Bolmgren & Eriksson, 2005; Gómez et al., 2016; Van der Niet et al., 2014). Additionally, floral and fruit traits reflect investments in resources by plants. Floral traits represent investments by plants to attract pollinators and ensure successful reproduction (Ashman & Schoen, 1996; Teixido et al., 2016). Similarly, fruit production represents a significant investment of resources to ensure successful seed dispersal and colonization, with plants allocating resources to produce fruits with various traits, balancing the costs and benefits based on environmental conditions and ecological contexts (Anderson & Hill, 2002). Therefore, floral and fruit functional traits represent the diverse array of plant reproductive strategies. These traits are often deemed highly stable within species (Cresswell, 1998), suggesting that their low intraspecific variability could mitigate methodological drawbacks associated with using mean trait values from databases. Also, flower and fruit traits are critical determinants of plant fitness by influencing pollination success, herbivore deterrence, seed dispersal, and resource allocation (Gavini et al., 2018; Stournaras & Schaefer, 2017). In this context, the functional traits of flowers and fruits possess significant yet underrated potential in functional research. However, their usability would require quantified verification, especially regarding the aspect of their variability.

To date, comparable studies have predominantly concentrated on assessing the traits of individual organs, quantifying the variability within a specific organ (Paź‐Dyderska & Jagodziński, 2024; Poorter et al., 2009; Snell et al., 2019). This prompted our exploration into the traits of three distinct organs, aiming to conduct a comparative analysis. In this current study, we chose to investigate the traits of underrepresented organs, namely, flowers and fruits, and compare them with the well‐established traits of leaves. Given that this represents one of the initial endeavors of its kind, we opted to exclusively focus on woody plants. This deliberate limitation aims to constrain potential sources of variability within the species pool. To minimize potential variability arising from environmental conditions, we chose to conduct the study in an Arboretum, where specimens grow in conditions similar to common garden settings, thus justifying the comparisons of trait measurements (Fanal et al., 2021; Faraji & Karimi, 2022; Perez et al., 2019).

We aimed to evaluate the variability of 16 functional traits (comprising six floral, six fruit, and four leaf traits) among a randomly selected group of woody species growing in comparable environmental conditions. Our focus was on assessing both interspecific and intraspecimen levels of variability (Liu et al., 2021; Westerband et al., 2021). Incorporating the intraspecific level of variation proved challenging in this study design. The Arboretum, despite its extensive collection, frequently lacks numerous specimens of the same species, with singular specimens being more common. Subsequently, we decided to check whether there are relationships among the interspecific variability of the traits studied, and their intracanopy variability represented by the plasticity index value. Lastly, we aimed to evaluate the potential of the proposed fruit and flower traits for future ecological research.

We hypothesized that (1) there would be a higher level of variation in floral and fruit traits at the interspecific level, given the generally more diversified forms of flowers and fruits compared to leaves among the subset of species studied here. Second, we hypothesized (2) an opposite pattern at the intraspecimen level, with fruits and flowers showing lower intraspecimen variability compared to leaves. This is because they are more evolutionarily predisposed to stability in form (Cresswell, 1998; van Kleunen et al., 2008), unlike leaves, which, as resource‐acquisition organs, exhibit higher plasticity and adaptability to changing light availability (Paź‐Dyderska et al., 2020; Poorter et al., 2009). Finally, we hypothesized that (3) floral and fruit traits would contribute new information to the global spectrum of plant form and function (Díaz et al., 2016; E‐Vojtkó et al., 2022), suggesting their potential usability in future ecological research.

## 2 METHODS

### 2.1 Study area

Our study was conducted within the Kórnik Arboretum in western Poland (52.2448° N, 17.0969° E, 75 m a.s.l.). Renowned as one of Poland's oldest and most extensive collections, it houses ca. 3500 taxa of woody plants, including numerous ornamental cultivars. Examining plant traits, especially in trees with extended maturation periods, requires consideration of plant age. The Arboretum's comprehensive dendrological collection renders it an appealing setting for such investigations. We have previously highlighted the advantages of utilizing the Arboretum for studies on the variability of specific leaf area (Paź‐Dyderska et al., 2020), floral chemical and size‐related traits (Paź‐Dyderska et al., 2022; Paź‐Dyderska & Jagodziński, 2024), and seed mass (Dylewski et al., 2024).

Historically, the Arboretum's management prioritized cognitive and ornamental purposes and was not explicitly designed for ecological studies. While some specimens were densely planted, others were isolated or arranged in small groups, forming alleys, potentially leading to uneven light distribution among individuals. Nonetheless, despite these considerations, the Arboretum stands out as one of the optimal choices for studying trait variability across a diverse range of species (Dosmann & Groover, 2012; Edwards et al., 2018; Fanal et al., 2021).

The study site is characterized by a temperate climate, featuring an average growing season lasting 220 days, a mean annual precipitation of 544 mm, and a mean annual temperature of 8.3°C. These climate parameters, documented in the Arboretum between 1948 and 2005 (Cedro & Iszkuło, 2011), set the backdrop for our research. In 2021 and 2022, during our data collection period, we gathered information from the meteorological station in the Arboretum, indicating mean annual precipitation and mean annual temperatures of 407 and 486 mm, and 9.9 and 10.1°C, respectively. The Arboretum offers consistent environmental conditions, encompassing both climate and soil type, providing an ideal setting for comparing traits across a diverse range of systematic and functional groups. This uniformity minimizes the influence of climate variations and extreme conditions, such as droughts, floods, or other disturbances, on the variability of the studied traits. Consequently, we assumed that comparing specimens growing in the Arboretum is justified, as the conditions in the Arboretum closely resemble a common garden design.

### 2.2 Data collection

We randomly selected a representative sample comprising 79 woody plant species cultivated under controlled common garden conditions (Figure S1), as outlined in our previous work (Paź‐Dyderska et al., 2020, 2022). Our data collection spanned from March 2021 to August 2022, emphasizing the examination of fully developed flowers, leaves, and fruits free from any damage caused by fungi, herbivores, or abiotic factors like hail or drought. Although collection protocols for floral or fruit traits are not yet widespread, we decided to incorporate established ideas from studies previously conducted in botanical gardens (Roddy, 2019; Roddy et al., 2016; Zhang et al., 2017). This methodology had been successfully employed in our previous investigation of floral traits within the Arboretum (Paź‐Dyderska et al., 2022; Paź‐Dyderska & Jagodziński, 2024). For leaves, we employed an approach adapted from Pérez‐Harguindeguy et al. (2013).

All organs were harvested at the zenith of their development phase, ensuring their complete maturation while preserving optimal conditions. To assess intra‐canopy trait variability, we gathered samples from two light regimes within each specimen, specifically targeting the shadiest and sunniest sections of the crown. This resulted in six samples for each specimen: two floral, two fruit, and two leaf samples. One sample from each pair originated from the sunny part, while the other came from the shady part of the specimen.

Consistent with our previous study in the Arboretum, we selected organs for the investigation based on qualitative observations, refraining from measuring light availability (Paź‐Dyderska et al., 2020, 2022). The collection process involved the use of a 6 m long pole pruner. To mitigate the influence of tree location on our results, we harvested samples exposed to sunlight from the southern sides of the crowns and shaded samples from the northern sides, resulting in the collection of two samples per specimen. Each sample typically consisted of 10 flowers, fruits, and leaves, respectively, resulting in a total of 20 flowers, fruits, and leaves per specimen. However, exceptions were made for species with exceptionally large flowers, such as *Magnolia tripetala*, where a smaller sample size was chosen to avoid potential harm to the valuable specimen. Adjustments were also made for species that did not produce a large number of fruits. Some species did not produce fruits, potentially influenced by year‐to‐year variability in environmental conditions such as late‐spring frost or summer drought. Additionally, the absence of a sufficient number of male specimens capable of fertilization could contribute to the lack of fruit production in some dioecious species. Furthermore, herbivory, particularly on fleshy fruits, was frequent, leading to many fruits being consumed by birds before reaching full ripening. As a result, samples of flowers and leaves were collected for 79 species, while fruits were obtained from 33 species, resulting in a total of 382 samples. This number is derived from the collection of double samples from each specimen, that is, samples from both light variants.

In terms of flower collection, for dioecious species, our exclusive focus was on female flowers since they transform into fruits, which are also the primary focus of our study. This rationale justified a comprehensive comparison of their traits. Throughout the sampling process, we systematically collected data on both the pedicel and peduncle, and for leaves, we included information on the petiole. After collection, we placed the samples in ziplock bags. This was done to minimize the risk of damage to the delicate samples and prevent moisture loss, which could potentially impact size‐related measurements. Subsequently, we stored the samples in a refrigerator to decelerate wilting, allowing them to await further trait measurements under optimal conditions.

### 2.3 Traits measurements

We examined a total of 16 traits for each sampled species, categorizing them into six floral, six fruit, and four leaf traits (Table 1). The floral traits comprised flower length (mm), width (mm), carbon content (%), nitrogen content (%), C:N ratio, and dry biomass (g). Similarly, fruit traits included fruit length (mm), diameter (mm), carbon content (%), nitrogen content (%), C:N ratio, and dry biomass (g). Leaf traits included specific leaf area (SLA, cm^2^ g^−1^), carbon content (%), nitrogen content (%), and C:N ratio. In our interspecific analyses, we calculated the mean values for each trait. For intracanopy analyses, we computed mean values from two samples collected from each specimen, specifically, from both the sunny and shaded parts of the specimen.

**TABLE 1:** Overview of studied traits and their biological significance.

Trait	Type of trait	Biological significance
Specific leaf area (cm^2^ g^−1^)	Morphological	Higher SLA values indicate thinner leaves with larger surface areas, enhancing photosynthetic efficiency and resource acquisition. SLA also reflects trade‐offs between growth rates and resource conservation. SLA influences plant defense mechanisms, environmental adaptation, and ecosystem functioning, shaping ecosystem processes such as carbon and nutrient cycling (Reich, 2014; Wright et al., 2004)
Leaf C (%)	Physiological	Leaf carbon content fuels the synthesis of essential organic compounds, supporting plant growth and development (Reich, 2014; Wright et al., 2004)
Leaf N (%)	Physiological	Higher leaf nitrogen content enhances photosynthetic rates and growth, contributing to increased productivity and plant resilience. It is also necessary for protein synthesis and enzyme activity. Leaf nitrogen content drives plant‐herbivore interactions and nutrient turnover rates (Díaz et al., 2016; Reich et al., 1998; Wright et al., 2004)
Leaf C:N (dimensionless)	Physiological	Optimal C:N ratios maintain nutrient balance, supporting vital processes like growth and reproduction. Deviations from this balance can signal nutrient limitations, impacting plant productivity. Additionally, C:N ratios influence plant quality as food for herbivores and decomposers, shaping nutrient cycling rates (Reich, 2014; Wright et al., 2004)
Flower C:N (dimensionless)	Physiological	This ratio reflects the allocation of carbon and nitrogen resources during flower development, influencing reproductive investment strategies and flower quality. Additionally, flower C:N ratios may influence flower defense mechanisms against herbivores and pathogens (Farré‐Armengol et al., 2013; Santiago & Sharkey, 2019)
Flower length (mm)	Morphological	Flower length influences the attraction of specific pollinators by accommodating their mouthparts and facilitating access to floral rewards. This trait promotes reproductive efficiency by enhancing pollen transfer between flowers and supporting cross‐pollination. Variation in flower length reflects coevolutionary dynamics with pollinators (E‐Vojtkó et al., 2022; Gómez et al., 2016)
Flower width (mm)	Morphological	Flower width influences the type of pollinators attracted to the flower, with wider flowers often accommodating bees, butterflies, and other pollinators seeking ample landing space and access to nectar and pollen. This trait impacts reproductive success by enhancing pollination efficiency and seed production (E‐Vojtkó et al., 2022; Gómez et al., 2016)
Flower dry biomass (g)	Morphological, physiological	Flower dry biomass reflects the resources allocated to flower production, with higher dry biomass indicating greater reproductive investment by the plant. Larger, more attractive flowers with higher dry biomass might attract more pollinators and achieve successful pollination, and seed set (Lussu et al., 2019; Teixido et al., 2016)
Flower C (%)	Physiological	Carbon serves as a structural component in flower tissues, contributing to their strength and durability. Flower carbon content also influences pollinator attraction and reward quality, as well as plant‐herbivore interactions, by affecting floral palatability and defense mechanisms (Heberling et al., 2019)
Flower N (%)	Physiological	Nitrogen is essential for flower development, determining pollen production. Flower nitrogen content affects pollinator attraction and reward quality. After pollination, nitrogen‐rich floral tissues contribute to soil nutrient pools through decomposition, supporting ecosystem productivity (Ma et al., 1997; Pers‐Kamczyc et al., 2020; Santiago & Sharkey, 2019)
Fruit length (mm)	Morphological	Fruit length influences seed dispersal mechanisms and survival. It mediates interactions between plants and frugivores, shaping seed dispersal patterns (Bentos et al., 2014; Stournaras & Schaefer, 2017)
Fruit diameter (mm)	Morphological	Fruit diameter impacts seed dispersal efficiency by influencing dispersal mechanisms and the preferences of frugivores, reflecting plant reproductive strategies (Bentos et al., 2014; Kourmpetli & Drea, 2014)
Fruit dry biomass (g)	Morphological, physiological	It reflects the investment of resources into fruit development, influencing reproductive success, and seed dispersal mechanisms. Additionally, fruit dry biomass contributes to the nutritional value of fruits for seed dispersers and consumers (Bentos et al., 2014; Kourmpetli & Drea, 2014)
Fruit C (%)	Physiological	Carbon‐rich fruits contribute to the structural integrity of the fruit, enhancing seed germination and seedling establishment. It also forms defensive compounds, such as phenols and tannins. That way, fruit carbon content influences interactions between plants and seed dispersers. It also contributes to nutrient cycling and soil fertility when fruits decompose (Feng et al., 2014; Rodríguez et al., 2013)
Fruit N (%)	Physiological	Nitrogen‐rich fruits provide essential nutrients for consumers, attracting a diverse range of frugivores and facilitating seed dispersal. After fruit consumption, the remaining nitrogen supports soil fertility and ecosystem productivity through decomposition (de Paz et al., 2018; Levey et al., 2000)
Fruit C:N (dimensionless)	Physiological	This ratio reflects the allocation of carbon and nitrogen resources during fruit development. It also affects seed dispersal strategies by shaping fruit attractiveness to seed dispersers (Birkhold et al., 1992; Garrido et al., 2023)

*Note*: Type of trait according to Violle et al. (2007).

We measured flower length, width, fruit length, and diameter using electronic calipers (with an accuracy of 0.001 mm) within 24 h of sample collection. Mean values were then calculated for each sample. Subsequently, we dried all flowers and fruits in an oven with forced air circulation at 65°C (ULE 600 and UF450, Memmert GmbH + Co. KG, Regensburg, Germany) until reaching a constant mass. We determined dry biomass using scales with an accuracy of 0.001 g.

We employed WinFOLIA 2020 PRO software (Regent Instruments Inc., Quebec, Canada) to scan leaves (along with the petiole) from each sample within 24 h of collection, measuring their area at a resolution of 300 DPI. Subsequently, we dried the leaves in an oven with forced air circulation at 65°C (ULE 600 and UF450, Memmert GmbH + Co. KG, Germany) until a constant mass was achieved. Following the drying process, we weighed the leaf samples using scales with an accuracy of 0.001 g. SLA, calculated as the ratio of leaf area to leaf dry biomass, was derived from these measurements.

Lastly, we assessed carbon and nitrogen content among the samples using an ECS CHNS − O 4010 Elemental Combustion System (Costech Instruments, Italy/USA) and a CHNS/O Analyser 2400 Series II (Perkin Elmer, USA).

### 2.4 Data analysis

For data analysis, we used the R software (R Core Team, 2023). All mean values were followed by standard errors (SE). For data processing, we used the dplyr package, and ggplot2 for visualization (Wickham, Chang, et al., 2020; Wickham, Francois, et al., 2020). To compare the variability of the studied traits at the interspecific level, we evaluated differences in trait coefficients of variation (CV, calculated as standard deviation divided by mean) between two traits using Krishnamoorthy and Lee's (2014) modified signed‐likelihood ratio test (M‐SLR test). This test was implemented through the cvequality::mslr_test() function (Marwick & Krishnamoorthy, 2019). We decided to use the M‐SLR test due to lower type I error rates and increased power across a range of conditions compared to the widely used asymptotic test of Feltz and Miller (1996), as noted by Funk (2008) and Paquette et al. (2012). Additionally, the M‐SLR test accommodates uneven sample numbers, a crucial feature for our study design. To avoid an increased risk of type I errors when performing multiple hypotheses tests (pairs of CVs for each trait), we also applied Holm‐Bonferroni corrections (Holm, 1979). Analyses involving interspecific variability were based on averaged trait values from both sun and shade parts of the crowns.

To compare the level of intraspecimen trait variability we used the plasticity index (PI). PI was calculated by evaluating the difference between maximum and minimum trait values, dividing it by the maximum trait values, following the methodology proposed by Valladares et al. (2000). In our study, the maximum and minimum values were obtained from the mean trait values in the two samples, each collected under sun and shade conditions. Consequently, the higher of the two values represented our maximum, and the lower represented our minimum. Analyses involving intracanopy trait variability comprised two samples (one from each light variant, i.e., sun or shade). We used PI for each species as a standardized measure of effect sizes, independent of trait values. For evaluating the impact of light variants on trait values, we employed a phylogenetic paired t‐test using the phyl.pairedttest function from the phytools package (Revell, 2020). In this *t*‐test we compared PI of each pair of traits, using species as data points. This test expands on the traditional paired *t*‐test by taking into account the phylogenetic similarity of observations. We acquired information about species phylogenetic similarity using a phylogenetic tree obtained from the V.PhyloMaker2 package (Jin & Qian, 2022). Similar to the interspecific analyses, here we also applied the Holm–Bonferroni correction (Holm, 1979).

Given our aim to compare the traits introduced in this study with the established traits constituting the global spectrum of plant form and function, we found it necessary to obtain data for the three traits not measured by us in the study (i.e., height, seed mass, and specific stem density) from the TRY database (Kattge et al., 2020). Since the TRY database did not provide values for these traits for all the studied species, we employed a data imputation approach to fill in the gaps. This method involved relying on correlations among traits and between traits and phylogeny, following a methodology used in our previous study (Paź‐Dyderska & Jagodziński, 2023) and consistent with the approach outlined by Pyšek et al. (2015). For imputation, we used known values of traits and phylogenetic eigenvectors, derived from the phylogenetic tree using the PVR package (Santos, 2018). These variables were used in trait‐wise random forest based imputations implemented in the missForest package (Stekhoven & Bühlmann, 2012). We decided to reinforce predictive power by phylogenetic eigenvectors as Penone et al. (2014) revealed that it can significantly increase accuracy of estimating missing trait values. By adopting this approach, we ensured a comprehensive dataset for analysis, thereby maintaining the integrity of our study despite the absence of values for some traits. We imputed missing flower traits data for seven species (8.8%), fruit traits for 46 species (58.2%), height for 15 species (19.0%), seed mass for 21 species (26.6%), and SSD for 49 species (62.0%). Normalized RMSE of imputation was 0.50. Such a proportion of missing data still allows for robust data analyses (Stewart et al., 2023).

To examine potential relationships among the newly proposed floral and fruit traits and well‐established traits within the global spectrum of plant form and function (Díaz et al., 2016), we performed Principal Component Analysis (PCA) using the vegan package (Oksanen et al., 2022). Using PCA allowed us to explore connections between various traits and evaluate whether the new traits represent additional dimensions of plant form and function variability among the studied species. Prior to PCA we standardized trait values by centering (i.e., mean subtraction) and scaling (i.e., dividing by standard deviation). We assessed bivariate relationships for each pair of studied traits following the method proposed by Zheng et al. (2009), using the corphylo function from the ape package (Paradis & Schliep, 2019).

## 3 RESULTS

### 3.1 Interspecific variability

**FIGURE 1:**
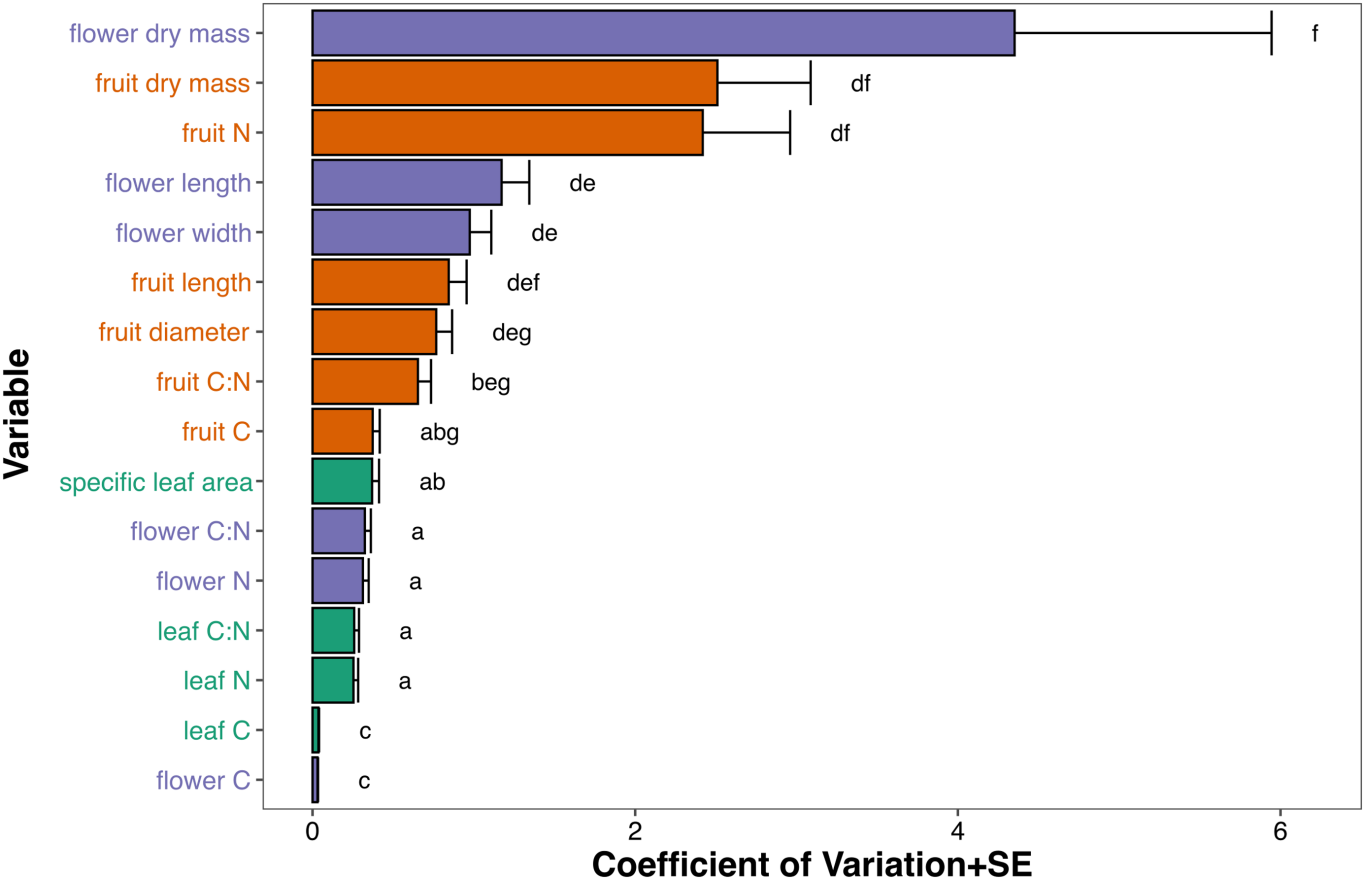
Mean + SE coefficient of variation (%) for the functional traits studied. Purple bars represent floral traits, orange bars – fruit traits, and green bars – leaf traits. Traits denoted with the same letter did not differ at *p* = .05 according to M‐SLR tests, after application of Holm's correction. For test statistics, see Table S1.

We observed that among the studied traits, those related to the dry mass of flowers and fruits had the highest interspecific variability, while traits associated with the carbon content in flowers and leaves had the lowest variability (Table 2, Figure 1). Specifically, flower dry biomass stood out with a CV = 4.351, showcasing the highest interspecific variability among the 16 analyzed traits. This was followed by fruit dry biomass (CV = 2.510) and fruit nitrogen content (CV = 2.419). Conversely, the least variable traits were those related to carbon. Size‐related traits, such as flower length and width, and fruit length and diameter, represented an intermediate level of variation. SLA demonstrated a limited level of interspecific variation (CV = 0.369), ranking below the variability of traits related to flower and fruit dry mass, as well as size‐related traits of flowers (CV of flower length = 1.172 and flower width = 0.974) and fruits (CV of fruit length = 0.844 and of diameter = 0.767).

**TABLE 2:** Species‐level averaged trait values.

Variable	Min	Q1	Median	Mean	SE	CV	Q3	Max
Specific leaf area (cm^2^ g^−1^)	34.100	171.636	206.255	215.486	8.949	0.369	249.043	565.605
Leaf C (%)	39.580	43.813	44.995	44.747	0.184	0.037	45.723	48.810
Leaf N (%)	1.115	1.890	2.245	2.221	0.063	0.254	2.513	4.405
Leaf C:N (dimensionless)	12.027	20.951	23.270	25.111	0.730	0.258	27.593	48.692
Flower C (%)	40.910	43.630	44.583	44.421	0.162	0.031	45.254	47.405
Flower N (%)	1.295	2.266	2.845	2.966	0.109	0.312	3.370	5.220
Flower C:N (dimensionless)	9.809	15.454	18.512	19.426	0.742	0.324	23.103	40.576
Flower length (mm)	0.681	8.196	14.026	20.655	2.723	1.172	21.048	155.679
Flower width (mm)	0.609	8.941	14.942	20.194	2.214	0.974	23.508	122.605
Flower dry biomass (g)	0.0001	0.005	0.037	0.241	0.118	4.351	0.116	9.166
Fruit length (mm)	3.710	9.964	15.918	24.341	3.577	0.844	36.833	89.419
Fruit diameter (mm)	2.869	7.392	9.257	13.509	1.803	0.767	14.320	45.710
Fruit dry biomass (g)	0.006	0.055	0.162	0.974	0.426	2.510	0.419	12.479
Fruit C (%)	0.553	45.840	47.035	41.986	2.726	0.373	48.535	53.065
Fruit N (%)	0.305	0.800	1.365	10.714	4.511	2.419	1.820	86.827
Fruit C:N (dimensionless)	16.954	38.776	46.245	57.495	6.538	0.653	72.341	232.402

Abbreviations: CV, coefficient of variation; Max, maximum; Min, minimum; Q1, first quartile; Q3, third quartile; SE, standard error.

### 3.2 Intraspecimen variability

**FIGURE 2:**
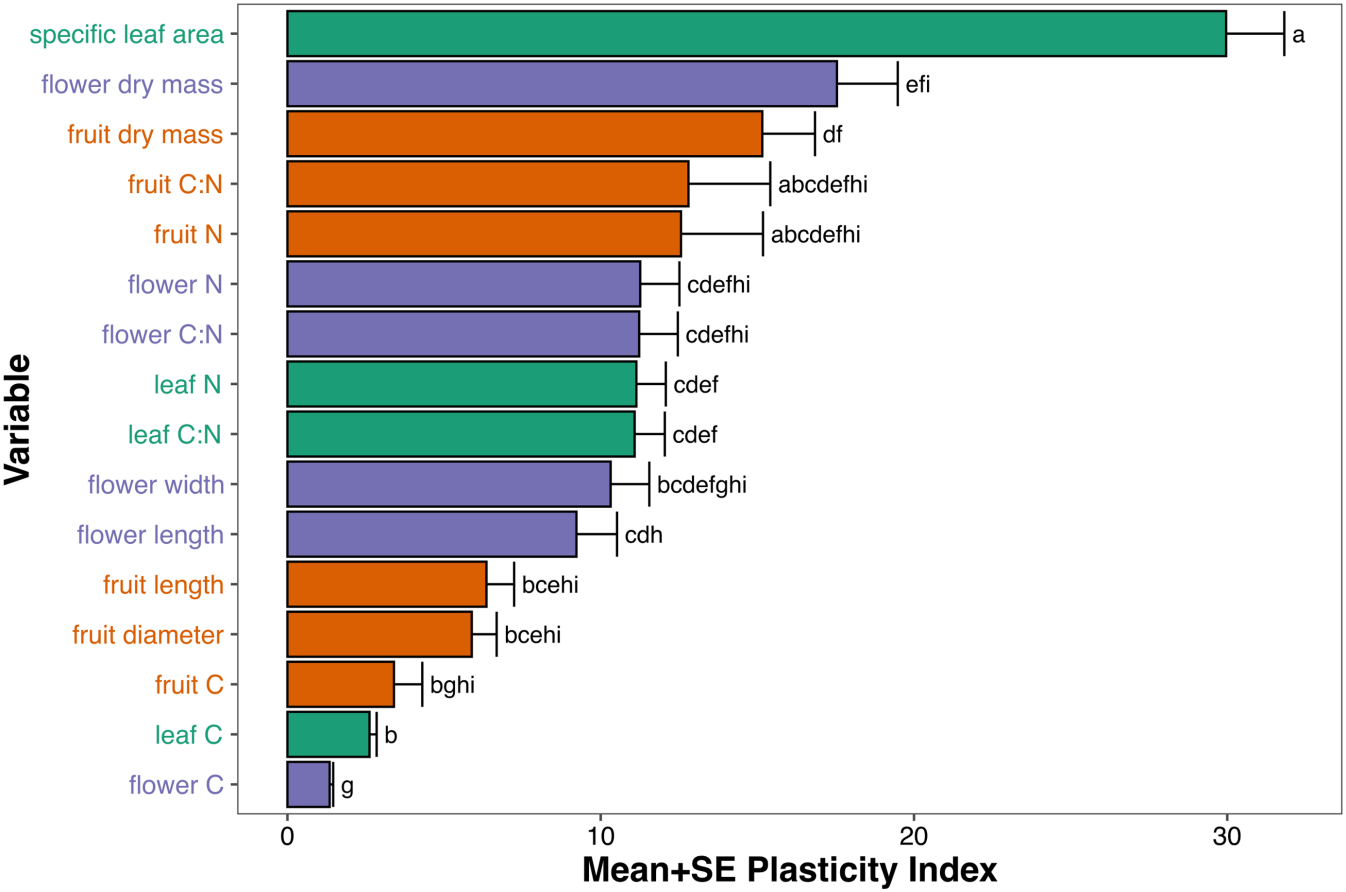
Mean + SE plasticity index (%) for the functional traits studied. Purple bars represent floral traits, orange bars – fruit traits, and green bars – leaf traits. Traits denoted with the same letter did not differ at *p* = .05, according to phylogenetic paired t‐test, after application of Holm's correction. For test statistics see Table S2.

Among the studied traits, SLA exhibited the highest variation within a specimen, with a mean PI of 29.963% (Table 3, Figure 2). This was followed by the dry biomass of flowers (PI = 17.541%) and fruits (PI = 15.158). In parallel to interspecific analyses, carbon‐content traits for all organs demonstrated the least variability, with flower carbon content being the least variable (PI = 1.347%). Nitrogen‐related traits represented intermediate levels of intracanopy variability (PI values for flowers, fruits, and leaves were 11.261%, 12.564%, and 11.139%, respectively). Similarly, size‐related traits showed values of 9.226% for flower length, 10.317% for flower width, 6.353% for fruit length, and 5.884% for fruit diameter.

**TABLE 3:** Plasticity index values (%).

Variable	Min	Q1	Median	Mean	SE	CV	Q3	Max
Specific leaf area (cm^2^ g^−1^)	1.054	17.294	30.792	29.963	1.859	0.551	41.103	69.340
Leaf C (%)	0.044	1.097	2.218	2.620	0.227	0.771	3.446	8.619
Leaf N (%)	0.000	5.239	8.955	11.139	0.939	0.749	14.329	38.760
Leaf C:N (dimensionless)	0.254	4.382	9.589	11.082	0.963	0.773	14.266	40.383
Flower C (%)	0.022	0.647	1.173	1.347	0.114	0.720	1.939	4.295
Flower N (%)	0.303	3.730	8.868	11.261	1.251	0.943	14.871	56.959
Flower C:N (dimensionless)	0.096	3.840	8.956	11.228	1.233	0.932	14.668	56.514
Flower length (mm)	0.189	3.050	5.851	9.226	1.295	1.248	9.949	72.728
Flower width (mm)	0.212	3.039	7.283	10.317	1.234	1.063	14.184	68.670
Flower dry biomass (g)	0.000	6.266	12.500	17.541	1.941	0.984	23.479	90.086
Fruit length (mm)	0.264	2.120	6.458	6.353	0.884	0.800	9.498	23.551
Fruit diameter (mm)	0.165	2.627	4.477	5.884	0.795	0.776	7.502	16.776
Fruit dry biomass (g)	0.971	6.742	13.606	15.158	1.682	0.638	24.379	35.103
Fruit C (%)	0.040	0.992	1.998	3.400	0.905	1.529	3.340	24.379
Fruit N (%)	0.000	1.408	9.333	12.564	2.617	1.197	16.770	64.444
Fruit C:N (dimensionless)	0.174	2.535	7.593	12.801	2.612	1.172	15.146	65.377

Abbreviations: CV, coefficient of variation; Max, maximum; Min, minimum; Q1, first quartile; Q3, third quartile; SE, standard error.

### 3.3 Interspecific versus intraspecimen variability of the traits studied

**FIGURE 3:**
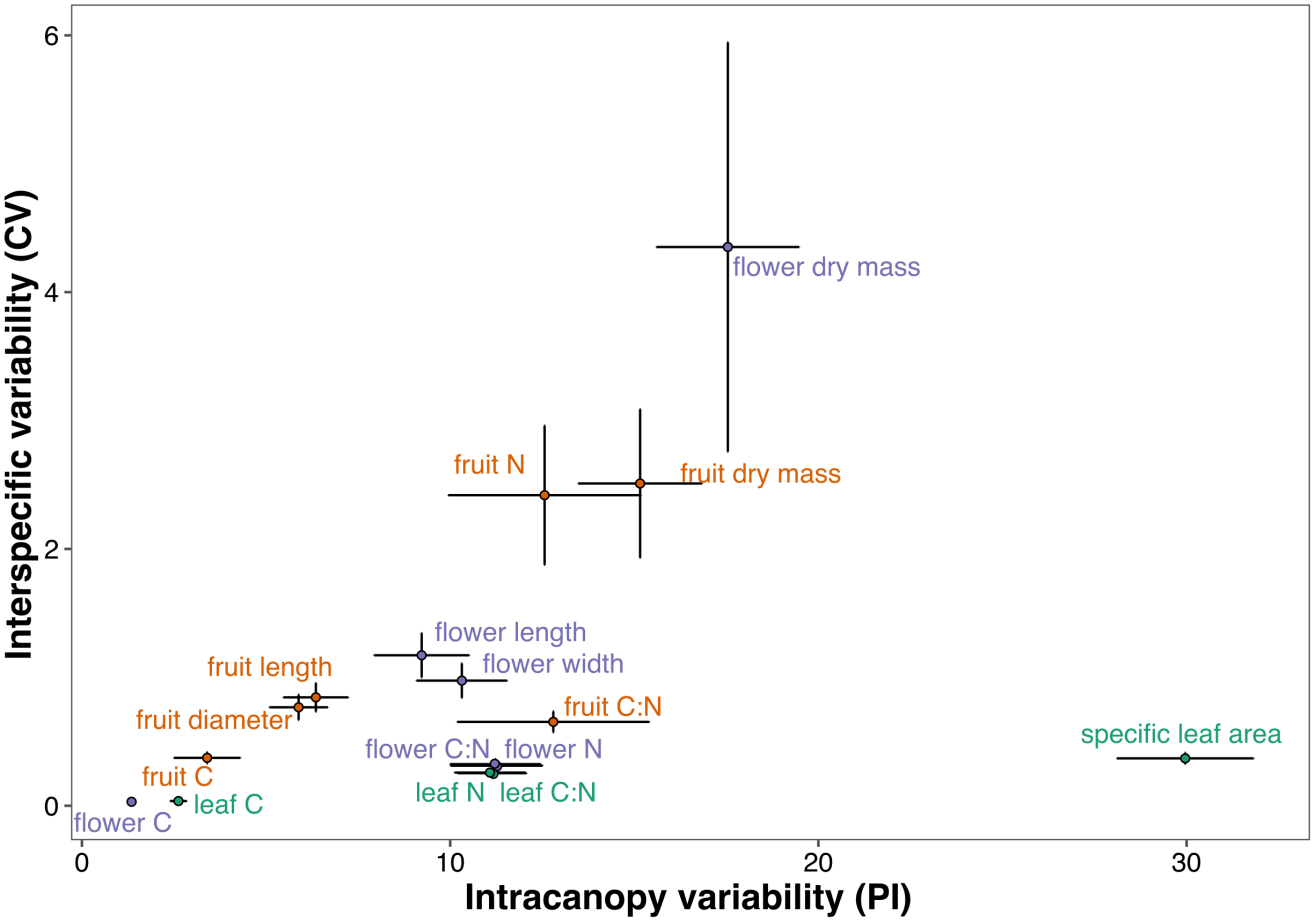
Comparison of mean ± SE interspecific trait variability, represented by the coefficient of variability (%) with the intracanopy variability of the respective traits, represented by the plasticity index (%) of a given trait. Purple labels represent floral traits, orange labels – fruit traits, and green labels – leaf traits.

In the visualized comparison of mean interspecific and intracanopy variability (Figure 3), we identified variations not only in the mean values of variability but also in their ranges. The traits reflecting carbon content in all three analyzed organs demonstrated the least variability, both in terms of interspecific and intracanopy variability. Traits related to size and nitrogen content across all organs exhibited intermediate values of coefficient of variation (CV) and plasticity index (PI). Flower dry mass stood out with the highest range of values representing interspecific variability and a limited range of values representing intracanopy variability. Notably, the position of SLA on the diagram showed particularly high mean intracanopy variability values, along with a wide range, while simultaneously exhibiting a low range of interspecific variability.

### 3.4 Relationships between established and novel traits proposed in this study

**FIGURE 4:**
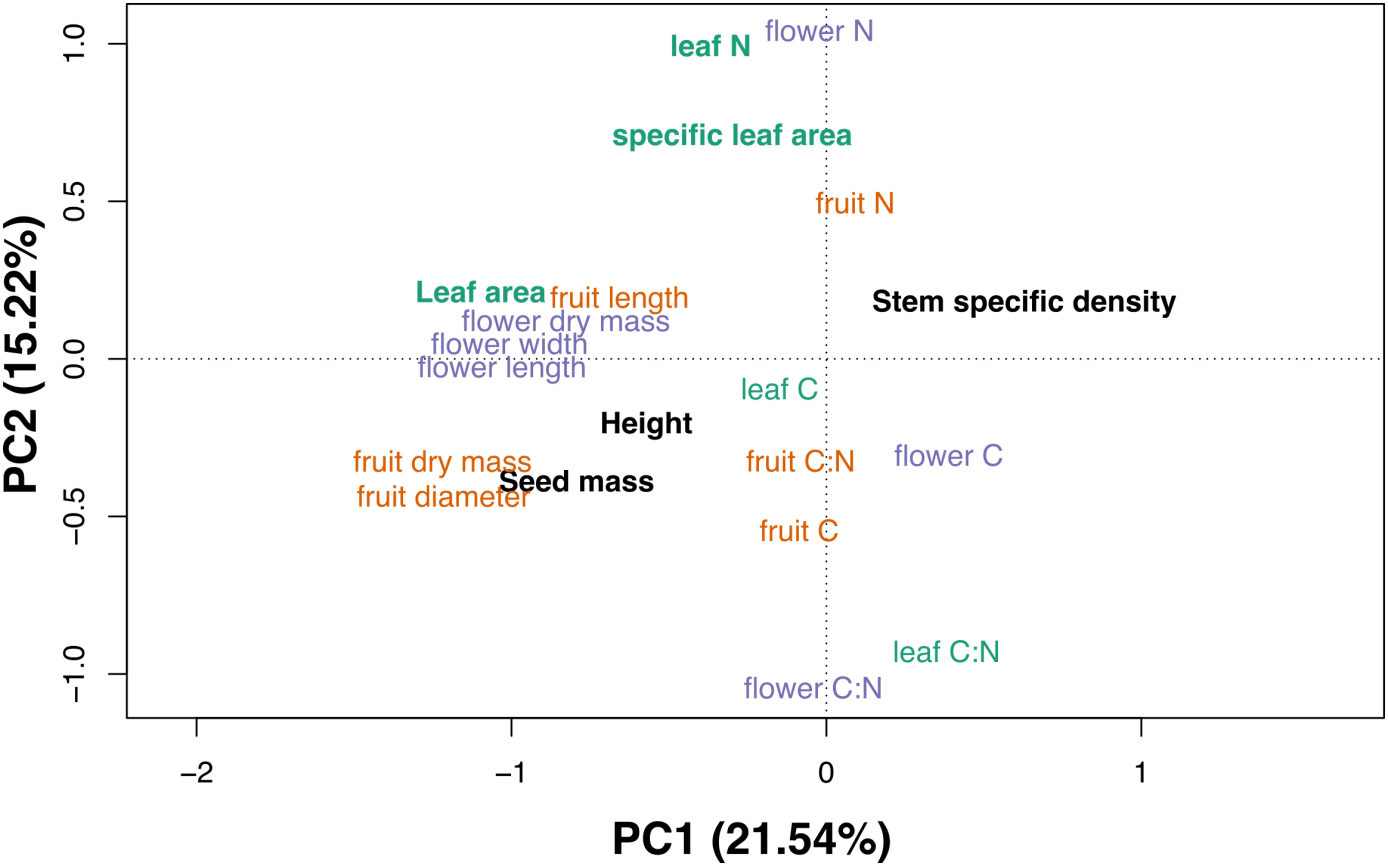
Results of principal components analysis comparing well‐established traits from the global spectrum of plant form and function (in bold) and lesser‐recognized traits proposed in this study. Purple labels represent floral traits, orange labels – fruit traits, and green labels – leaf traits. Black labels indicate traits from the global spectrum of plant form and function, values of which were derived from the TRY database (see Section 2.4) (Kattge et al., 2020).

**FIGURE 5:**
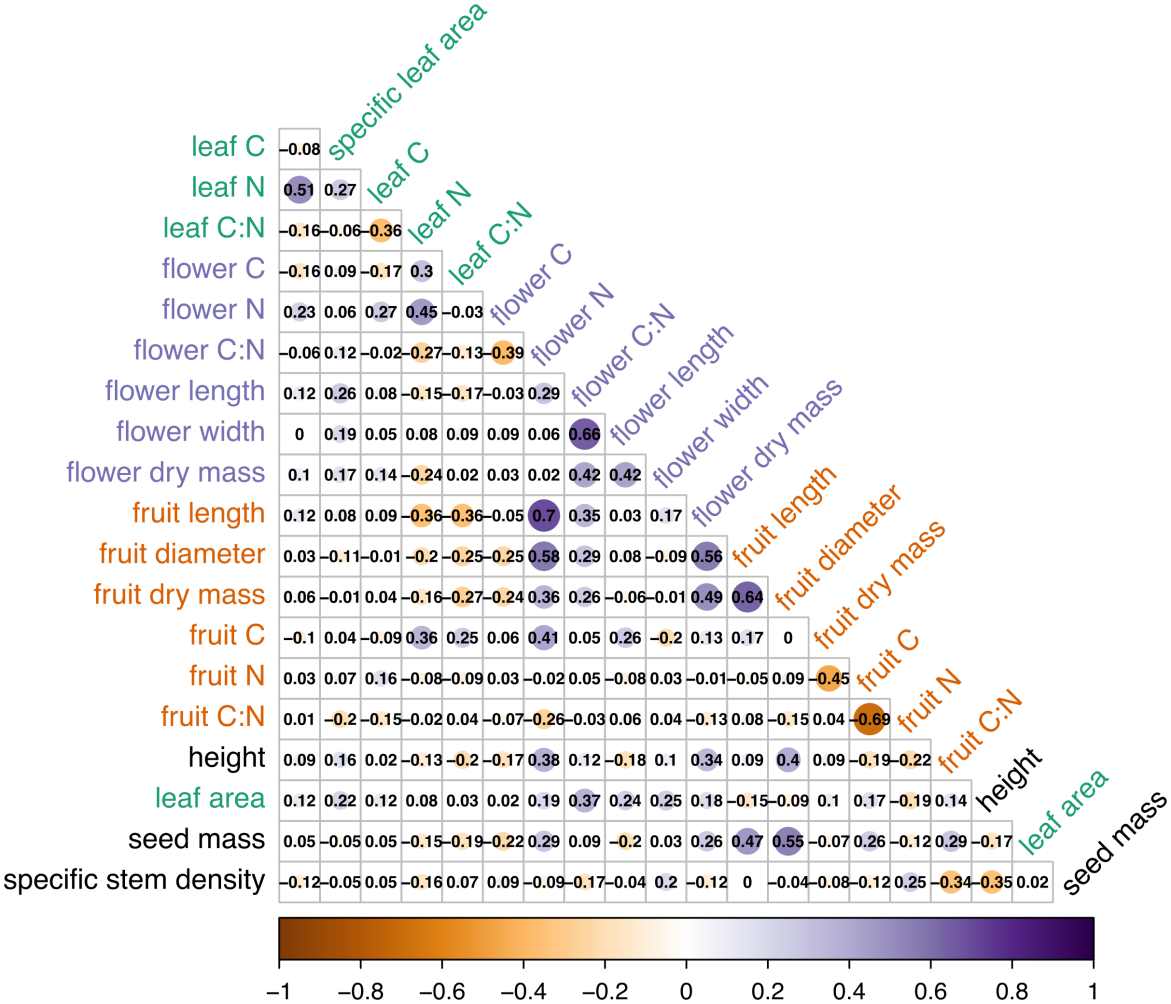
Correlation matrix describing the strength of bivariate relationships between each pair of traits, calculated accounting for phylogenetic dependence of species via the corphylo function from the ape package (Paradis & Schliep, 2019), expressed as Pearson's correlation coefficients (*r*). Purple labels represent floral traits, orange labels – fruit traits, and green labels – leaf traits. Black labels indicate traits from the global spectrum of plant form and function, values of which were derived from the TRY database (see Section 2.4) (Kattge et al., 2020).

The PCA revealed the main directions of variation (Figure 4), with the PC1 axis explaining 21.54% of the variability and the PC2 axis explaining 15.22% of the variability. Negative values on the PC1 axis were associated with higher values of three traits proposed by Díaz et al. (2016), namely leaf area, height, and seed mass, along with higher values of mass‐ and size‐related traits of flowers and fruits. Conversely, positive values on the PC1 axis were correlated with specific seed density, which increased proportionally with the values on the PC1 axis. Increasing values on the PC2 axis were correlated with higher nitrogen concentration in all three analyzed organs, as well as increasing SLA. Conversely, the PC2 axis showed a negative correlation with carbon concentration and C:N ratio, regardless of the organ analyzed. None of traits measured by us revealed a separate dimension of variability in trait space. Analysis of bivariate correlations revealed the strongest relationships among traits expressing organ sizes (e.g., fruit length and diameter), as well as C:N ratios and N contents (Figure 5). Seed mass was positively correlated with fruit dimensions and dry mass and height.

## 4 DISCUSSION

### 4.1 Interspecific variability of the traits studied

Among the traits we examined, the most noticeable differences among species manifested within traits related to the dry biomass of flowers and fruits, while the most stable traits pertained to carbon content in flowers and leaves. The significant heterogeneity in the dry mass of flowers and fruits across the surveyed species can be attributed to the adaptation to diverse pollination and dispersal mechanisms. The broad diversity of flower forms, which includes not only morphological structures but also dimensions, can result from various selection agents, including selective herbivores, abiotic factors, mutualistic interactions with other organisms such as fungi, and, to a significant extent, varied pollination factors (Chanderbali et al., 2016; Chartier et al., 2021). For instance, the categorization of species into insect‐ and wind‐pollinated explains the observed diversity (Armbruster et al., 2000; Sapir et al., 2019). Wind‐pollinated species, for example oaks or pines, do not have a justification to expend resources on the costly production of large‐volume, distinct flowers, given their high abundance of airborne pollen. Conversely, species engaged in competition for pollinators, such as *Magnolia* spp. or *Cornus* spp., necessitate more showy or fragrant flowers to be more attractive than competing species, thereby driving further diversification in morphological structures of flowers.

Similarly, concerning fruits, whose dimensions often correlate with those of flowers, their fundamental role remains consistent: to facilitate the successful reproduction of the specimen (Kourmpetli & Drea, 2014). However, the strategies employed to realize this objective vary markedly among species. Some fruits, as in willows, necessitate reduction in mass for anemochoric dispersal, while others require substantial mass to benefit from the proximity to the maternal tree, or from the resources provided, as in *Aesculus* spp. or *Quercus* spp. Furthermore, the structural composition of fruits, whether categorized as dry or fleshy, is intrinsically linked to the species' reproductive strategy. Fleshy fruits, for instance, serve to attract herbivores and facilitate seed dispersal by endozoochory, often concurrently providing essential resources for the seed after dispersion through animal excretion, as in *Prunus* spp. Conversely, dry fruits predominantly serve as a protective barrier for the seed against external agents, given that the seed frequently already harbors the requisite resources for germination, as in *Juglans* spp. The expansive array of pollination mechanisms and reproductive strategies constitutes a plausible rationale for the considerable variability observed in flower and fruit dry biomass.

As species with different ecological strategies can exhibit different carbon allocation patterns (Grasset et al., 2015; Trugman et al., 2019), we might expect varying carbon allocation patterns in flowers, fruits, and leaves across the species analyzed. However, in our study, we observed minimal variability of carbon content across these three organs. When examining the stability of traits linked to carbon content in plant organs, we can attribute it to the fundamental role of carbon as a building block. Carbon forms the basis of the main structural components of plant cells. For example, cellulose, the primary component of plant cell walls, consists of carbon, hydrogen, and oxygen (McFarlane et al., 2014). Similarly, lignin, another crucial structural element, provides strength to cell walls and also contains carbon (Neutelings, 2011). As a result, the consistent presence of carbon in plant organs displays limited variability.

In contrast, nitrogen content, especially in fruits, exhibits a higher level of variability compared to carbon. This variability can be attributed to the pivotal role of nitrogen in amino acids, the essential building blocks of proteins (Atilio & Causin, 1996). Since nitrogen is a critical nutrient for plants, its deficiency triggers a series of physiological and biochemical responses aimed at efficient nitrogen acquisition and utilization (Ye et al., 2022). These responses include reduced growth (Broadley et al., 2000), delayed development (Ma et al., 1997), and changes in the allocation of resources (Mu & Chen, 2021). In response to nitrogen deficiency, plants strategically redistribute resources, prioritizing essential functions (Zhao et al., 2005). For instance, nitrogen is reallocated from older leaves to younger, actively growing tissues, ensuring that the limited nitrogen is directed toward critical physiological processes (Ueda et al., 2011). This dynamic response highlights the plant's adaptability in optimizing resource utilization under varying nitrogen conditions.

### 4.2 Intraspecimen variability of the traits studied

At the intraspecimen level, our investigation highlighted SLA as the most variable trait, contrasting with the overall stability observed in carbon content across all three examined organs, consistent with interspecific analyses. While prior studies have acknowledged the high variability of SLA within individual specimens (Gunn et al., 1999; Paź‐Dyderska et al., 2020), it is essential to underscore the substantial difference in its variability compared to other traits tested. SLA is a valuable indicator of resource availability. Therefore, it can vary significantly even within a single specimen. This dynamic adaptability of SLA stems from leaves adjusting to varying light availability, leading to observable changes in SLA values (Legner et al., 2014; Poorter et al., 2006; Reich et al., 1998).

Leaves, functioning as the primary assimilation apparatus of plants, play a pivotal role in photosynthesis, utilizing light, carbon dioxide, and water to synthesize glucose and other carbohydrates (Crang et al., 2018). These carbohydrates not only act as an energy source for diverse plant activities but also serve as fundamental building blocks for various organic compounds. Consequently, changes in light availability prompt adaptive responses in SLA, optimizing resource utilization (Gratani et al., 2006; James & Bell, 2000; Wyka et al., 2012). In contrast, flowers and fruits, reliant on the photosynthetic products generated by leaf activity, may not exhibit as pronounced a response to changes in light availability (but see Garrido et al., 2023). Their stability in the face of varying light conditions can be attributed to their dependency on the consistent products of photosynthesis provided by leaves. It is worth noting that the potential impact of light availability on flowers might be further diminished by considering that several species blossom before the development of leaves (Tal, 2011).

Yet, we have still identified significant intracanopy variability for both flowers and fruits, with flower and fruit dry biomass emerging as the second and third most variable traits in our analysis. This variability could be ascribed to intentional placement strategies employed by woody species for their flowers and fruits within the canopy. The deliberate positioning of flowers is especially vital due to its key role in ensuring successful pollination, subsequently influencing fruit and seed production (Khalil et al., 2023). Additionally, the location of flowers can be influenced by the imperative need to attract specific pollinators (Damascena et al., 2017). Some trees place their flowers in visible and accessible positions to enhance the probability of attracting pollinators.

Similarly, the positioning of fruits within the canopy contributes to intracanopy variability of various fruit parameters. For example, light exposure can significantly affect fruit quality, as demonstrated in various *Malus* × *domestica* cultivars. In apples, fruits from the outer canopy exhibited higher fresh weight and soluble solids content compared to those from the inner canopy. Additionally, the outer‐canopy fruits had higher concentrations of soluble sugars and sugar alcohols, but lower starch concentrations (Feng et al., 2014). Nilsson and Gustavsson (2007) similarly found that fruits developing outside the canopy acquired a red peel color during maturation, while those inside remained green. Outside fruits had higher contents of dry matter, soluble solids, and soluble sugars, but lower amounts of titratable acidity than inside fruits. According to the results of Tustin et al. (1988), both fresh weight and soluble solids concentration showed highly positive correlations with the transmission of photosynthetic photon flux. Consequently, fruit fresh weight and soluble solids concentration increased with increasing height in the canopy and were higher in the outer canopy position.

Intracanopy variability in fruits may arise because those in the upper canopy receive more direct sunlight, promoting enhanced photosynthesis and facilitating optimal fruit ripening (Khalil et al., 2023). This is particularly crucial for tree species reliant on sunlight for the initiation and completion of the ripening process, such as trees of *Malus* spp. or *Prunus* spp. Moreover, some trees employ a strategy of placing fruits at higher positions within the canopy to capitalize on wind dispersal (Qin et al., 2022). Elevated fruits are more likely to be caught by the wind, facilitating their dispersal away from the parent tree and increasing the likelihood of successful seed dispersal and colonization in new areas (Qin et al., 2022). While these active strategies employed by trees and shrubs underscore the variability observed in flower and fruit traits in our study, a comprehensive understanding of the factors influencing such variations would require further studies.

### 4.3 Potential of the floral and fruit traits in functional ecology

In examining the potential of floral and fruit traits in functional ecology, our study establishes connections between the newly proposed traits and the widely recognized traits delineated by Díaz and co‐authors (Díaz et al., 2016). Employing six traits from the global spectrum of plant form and function, our PCA revealed two axes of variation within the species pool: one reflecting a size‐related variability of traits and the second, explaining variability in chemical composition of the studied plants.

While our novel floral and fruit traits don't introduce new dimensions to the variability already explained by the six traits proposed by Díaz et al. (2016), their significance lies in their alignment with the established traits. This alignment underscores the potential for predicting floral and fruit traits based on the well‐recognized traits from Díaz et al. (2016). For instance, our results suggest that species with high leaf area and seed mass values are likely to exhibit flowers and fruits with higher dry biomass and larger size. Also, based on the analysis of bivariate correlations, we observed a positive correlation between seed mass, fruit diameter, and fruit dry biomass. However, these results contrast with those of Bentos et al. (2014), who identified a positive correlation between the number of seeds per fruit and fruit mass, but a negative correlation with seed mass. This disparity may be attributed to the different life strategies exhibited by the species studied, particularly since the discussed study focused on pioneer tree species of the Amazon forest. In our results, we also found a positive correlation between seed mass and height, consistent with the well‐established findings of Díaz et al. (2016). Notably, the size‐related traits of both flowers and fruits showed significant overlap, indicating a potential correlation between the sizes of these organs. This idea could be supported by early work on the topic of coordination between traits related to organ or organism size (Ackerly & Donoghue, 1998; Bond & Midgley, 1988; Corner, 1949), often referred to as Corner's rules. In contrast, SLA did not align as strongly within the size‐related traits, possibly due to its robust correlation with leaf nitrogen content, a relationship well‐documented in prior studies (Díaz et al., 2016; Reich et al., 1998; Wright et al., 2004).

In summary, while our novel traits don't contribute new dimensions to the understanding of plant form and function variability, they align effectively within existing dimensions. This alignment offers the potential to predict floral‐ and fruit‐related traits, and consequently, plant reproductive strategies, based on more established and readily available plant functional traits. However, this predictive capacity necessitates further studies incorporating data from a broader array of species.

### 4.4 Study limitations

During the initial phase of our study, we carefully weighed trade‐offs that introduced particular limitations. To ensure a broader diversity of species in our research, we opted to include only one representative specimen per species. While there is no universally determined optimal sample size for studying the intraspecific variability of flowers, existing research on foliar traits suggests that the most accurate sampling size involves four samples from 10 individuals (Petruzzellis et al., 2017). Despite recognizing that a larger number of specimens would offer a more comprehensive representation of data variability, we chose to concentrate on collecting flowers from individual specimens. This decision was influenced not only by the Arboretum's layout, where frequently only one mature specimen of each species is present, but also by the labor‐intensive nature of data collection.

The dynamic nature of flowering, occurring at various times throughout the growing season, rendered it impractical to collect data at a single time point, unlike leaves that remain accessible for months during the vegetative season. Additionally, we acknowledge that flowers, akin to other plant organs, undergo seasonal variations in their chemical composition, encompassing both the C and N contents analyzed in our study and other compounds (Ernst et al., 1991; Kenis et al., 1985; McMann et al., 2022). Nevertheless, our investigation stands as one of the pioneering efforts to explore the potential of C and N content in flowers for applications in the field of functional ecology. As a result, we consciously chose to temporarily set aside consideration of this type of variability, deeming it of minimal significance at this stage of the field's development. To standardize results we decided to sample organs of each species in the optimum of their development.

We encountered challenges in collecting fruits from the specimens under study, as certain species did not produce fruits. As detailed in the Section 2, this phenomenon could be attributed to the year‐to‐year variability in environmental conditions or the insufficient number of male specimens capable of fertilization in certain dioecious species. Additionally, herbivory was prevalent, leading to the consumption of many fruits by birds before reaching full ripening. Despite these challenges, as one of the pioneering studies of its kind, we believe that the innovative nature of the research can contribute to addressing specific limitations. Nevertheless, we emphasize that further studies, incorporating data not only from a greater variety of species but also from numerous specimens of the same species, are crucial for a more in‐depth exploration of intraspecific trait variability of reproductive organs.

## 5 CONCLUSIONS

Our objective was to contribute to the dynamic field of functional ecology, specifically in relation to reproductive organs – flowers and fruits. To achieve this, we introduced floral and fruit traits that had been previously overlooked, employing a quantitative approach to assess their variability. Our investigation involved exploration of the variability and stability of these newly introduced traits, concentrating on 12 quantitative traits that offer insights into the reproductive strategies of woody species. Despite the development of studies on floral and fruit functional traits over the last years, comparative analyses of the variability in these traits with established ones are rare, if not absent. Through our analysis, we unveiled significant interspecific variability across these traits, exposing a broad spectrum of diversity among the studied species. We delved into intraspecific differences, revealing a limited level of variation within individual specimens. Furthermore, we investigated correlations between these traits and widely utilized traits from the global spectrum of plant form and function proposed by Díaz et al. (2016), uncovering intriguing connections between the established traits and the floral and fruit traits upon which we focused, and complementing the already well‐recognized variability in plant form and function with additional insights into reproductive processes.

## AUTHOR CONTRIBUTIONS

**Sonia Paź‐Dyderska:** Conceptualization (equal); data curation (lead); formal analysis (lead); funding acquisition (equal); investigation (lead); methodology (equal); project administration (lead); software (lead); validation (lead); visualization (lead); writing – original draft (lead). **Andrzej M. Jagodziński:** Conceptualization (equal); funding acquisition (equal); methodology (equal); supervision (lead); writing – review and editing (lead).

## FUNDING INFORMATION

The study was financed by the National Science Centre, Poland, as part of Project No. 2020/37/N/NZ8/00387 titled: “How to better describe the functioning of species in an ecosystem? Reproduction traits and their potential in functional ecology of plants.” The study was partially supported by the Institute of Dendrology of the Polish Academy of Sciences.

## CONFLICT OF INTEREST STATEMENT

The authors declare no conflict of interest.

## Supporting information


Appendix S1.

## Data Availability

The data that support the findings of this study are openly available in figshare at https://doi.org/10.6084/m9.figshare.25204934.v1.
