# Supplementary material for: Potential of reproductive traits in functional ecology: A quantitative comparison of variability in floral, fruit, and leaf traits
Source: Ecol Evol. 2024 Jul 18;14(7):e11690. doi: 10.1002/ece3.11690 (PMC11255459; doi:10.1002/ece3.11690)
Supplement: Supplementary file 1 — Appendix S1. [file ECE3-14-e11690-s001.docx]

**Table S1.** Results of trait-wise Krishnamoorthy and Lee’s (2014) modified signed-likelihood ratio tests. Upper part – test statistics, lower part – p-values.

|  | **SLA** | **Leaf C** | **Leaf N** | **Leaf C:N** | **Flower C** | **Flower N** | **Flower C:N** | **Flower length** | **Flower width** | **Flower dry mass** | **Fruit length** | **Fruit diameter** | **Fruit dry mass** | **Fruit C** | **Fruit N** | **Fruit C:N** |
| --- | --- | --- | --- | --- | --- | --- | --- | --- | --- | --- | --- | --- | --- | --- | --- | --- |
| **SLA** | - | 260.114 | 8.113 | 8.323 | 289.565 | 1.695 | 0.932 | 47.300 | 38.414 | 96.755 | 19.636 | 16.455 | 59.589 | 0.009 | 58.869 | 11.675 |
| **Leaf C** | <0.001 | - | 187.414 | 186.341 | 2.151 | 232.275 | 225.953 | 392.314 | 356.026 | 399.808 | 297.513 | 312.805 | 412.056 | 218.084 | 395.157 | 282.589 |
| **Leaf N** | 0.278 | <0.001 | - | 0.084 | 210.497 | 2.946 | 3.819 | 87.185 | 77.979 | 147.600 | 45.344 | 40.316 | 88.414 | 6.156 | 98.742 | 32.802 |
| **Leaf C:N** | 0.197 | <0.001 | 1.000 | - | 201.835 | 2.223 | 3.064 | 80.030 | 71.894 | 165.882 | 50.333 | 43.256 | 101.514 | 5.501 | 94.632 | 29.184 |
| **Flower C** | <0.001 | 1.000 | <0.001 | <0.001 | - | 201.766 | 222.964 | 371.993 | 344.968 | 391.629 | 272.082 | 291.973 | 359.149 | 205.748 | 365.052 | 295.005 |
| **Flower N** | 1.000 | <0.001 | 1.000 | 1.000 | <0.001 | - | 0.060 | 59.089 | 48.346 | 107.254 | 27.501 | 24.128 | 78.382 | 0.984 | 86.744 | 18.762 |
| **Flower C:N** | 1.000 | <0.001 | 1.000 | 1.000 | <0.001 | 1.000 | - | 55.787 | 50.686 | 101.036 | 26.812 | 23.748 | 75.836 | 0.640 | 78.306 | 14.346 |
| **Flower length** | <0.001 | <0.001 | <0.001 | <0.001 | <0.001 | <0.001 | <0.001 | - | 0.804 | 13.381 | 1.598 | 2.804 | 3.642 | 26.065 | 3.666 | 6.355 |
| **Flower width** | <0.001 | <0.001 | <0.001 | <0.001 | <0.001 | <0.001 | <0.001 | 1.000 | - | 21.394 | 0.422 | 1.130 | 7.334 | 17.215 | 6.323 | 3.125 |
| **Flower dry mass** | <0.001 | <0.001 | <0.001 | <0.001 | <0.001 | <0.001 | <0.001 | 0.034 | 0.003 | - | 9.735 | 12.544 | 0.496 | 46.881 | 0.582 | 15.545 |
| **Fruit length** | 0.001 | <0.001 | <0.001 | <0.001 | <0.001 | <0.001 | <0.001 | 1.000 | 1.000 | 0.177 | - | 0.137 | 6.393 | 11.048 | 6.968 | 0.981 |
| **Fruit diameter** | 0.005 | <0.001 | <0.001 | <0.001 | <0.001 | <0.001 | <0.001 | 1.000 | 1.000 | 0.016 | 1.000 | - | 9.296 | 9.276 | 8.696 | 0.437 |
| **Fruit dry mass** | <0.001 | <0.001 | <0.001 | <0.001 | <0.001 | <0.001 | <0.001 | 1.000 | 0.500 | 1.000 | 0.327 | 0.197 | - | 35.227 | 0.081 | 12.623 |
| **Fruit C** | 1.000 | <0.001 | 0.897 | 1.000 | <0.001 | 1.000 | 1.000 | <0.001 | 0.002 | <0.001 | 0.027 | 0.125 | <0.001 | - | 33.070 | 6.838 |
| **Fruit N** | <0.001 | <0.001 | <0.001 | <0.001 | <0.001 | <0.001 | <0.001 | 1.000 | 0.624 | 1.000 | 0.654 | 0.189 | 1.000 | <0.001 | - | 13.231 |
| **Fruit C:N** | 0.122 | <0.001 | <0.001 | <0.001 | <0.001 | 0.002 | 0.003 | 0.981 | 1.000 | 0.004 | 1.000 | 1.000 | 0.038 | 0.752 | 0.036 | - |

**Table S2.** Results of phylogenetic paired t-tests for traits PI, applied for each pair of traits. Upper part – test statistics, lower part – p-values.

|  | **SLA** | **Leaf C** | **Leaf N** | **Leaf C:N** | **Flower C** | **Flower N** | **Flower C:N** | **Flower length** | **Flower width** | **Flower dry mass** | **Fruit length** | **Fruit diameter** | **Fruit dry mass** | **Fruit C** | **Fruit N** | **Fruit C:N** |
| --- | --- | --- | --- | --- | --- | --- | --- | --- | --- | --- | --- | --- | --- | --- | --- | --- |
| **SLA** | - | 13.680 | 9.072 | 8.360 | 10.081 | 9.430 | 9.404 | 9.014 | 6.175 | 4.236 | 7.294 | 7.036 | 4.551 | 7.658 | 3.864 | 3.854 |
| **Leaf C** | <0.001 | - | -8.897 | -8.625 | 5.491 | -6.874 | -6.956 | -5.049 | -3.247 | -7.469 | -3.622 | -1.815 | -7.380 | -0.389 | -3.810 | -3.902 |
| **Leaf N** | <0.001 | <0.001 | - | 0.320 | 9.904 | 0.019 | 0.038 | 1.126 | -0.519 | -2.712 | 3.278 | 3.723 | -1.383 | 4.554 | -0.191 | -0.259 |
| **Leaf C:N** | <0.001 | <0.001 | 1.000 | - | 9.500 | -0.034 | -0.016 | 1.054 | -0.557 | -2.735 | 3.202 | 3.749 | -1.320 | 4.473 | -0.161 | -0.237 |
| **Flower C** | <0.001 | <0.001 | <0.001 | <0.001 | - | -7.869 | -7.970 | -5.442 | -3.111 | -8.497 | -6.188 | -4.636 | -8.645 | -2.356 | -4.320 | -4.405 |
| **Flower N** | <0.001 | <0.001 | 1.000 | 1.000 | <0.001 | - | 0.199 | 1.689 | -0.337 | -2.970 | 1.989 | 2.518 | -2.296 | 3.505 | -0.309 | -0.407 |
| **Flower C:N** | <0.001 | <0.001 | 1.000 | 1.000 | <0.001 | 1.000 | - | 1.688 | -0.382 | -3.004 | 2.033 | 2.546 | -2.262 | 3.536 | -0.288 | -0.386 |
| **Flower length** | <0.001 | 0.001 | 1.000 | 1.000 | <0.001 | 1.000 | 1.000 | - | -1.663 | -4.041 | 0.884 | 1.243 | -3.366 | 2.466 | -1.437 | -1.519 |
| **Flower width** | <0.001 | 0.243 | 1.000 | 1.000 | 0.363 | 1.000 | 1.000 | 1.000 | - | -3.302 | 0.952 | 1.303 | -3.612 | 2.617 | -1.740 | -1.821 |
| **Flower dry mass** | 0.011 | <0.001 | 0.958 | 0.915 | <0.001 | 0.500 | 0.475 | 0.021 | 0.211 | - | 2.963 | 3.118 | 0.081 | 4.029 | 0.653 | 0.619 |
| **Fruit length** | <0.001 | 0.160 | 0.360 | 0.419 | <0.001 | 1.000 | 1.000 | 1.000 | 1.000 | 0.709 | - | 0.494 | -6.822 | 2.405 | -2.509 | -2.610 |
| **Fruit diameter** | <0.001 | 1.000 | 0.123 | 0.117 | 0.012 | 1.000 | 1.000 | 1.000 | 1.000 | 0.496 | 1.000 | - | -7.802 | 2.080 | -2.612 | -2.740 |
| **Fruit dry mass** | 0.014 | <0.001 | 1.000 | 1.000 | <0.001 | 1.000 | 1.000 | 0.291 | 0.162 | 1.000 | <0.001 | <0.001 | - | 6.562 | 0.852 | 0.771 |
| **Fruit C** | <0.001 | 1.000 | 0.014 | 0.018 | 1.000 | 0.214 | 0.203 | 1.000 | 1.000 | 0.058 | 1.000 | 1.000 | <0.001 | - | -3.130 | -3.229 |
| **Fruit N** | 0.089 | 0.100 | 1.000 | 1.000 | 0.028 | 1.000 | 1.000 | 1.000 | 1.000 | 1.000 | 1.000 | 1.000 | 1.000 | 0.489 | - | -0.587 |
| **Fruit C:N** | 0.090 | 0.081 | 1.000 | 1.000 | 0.022 | 1.000 | 1.000 | 1.000 | 1.000 | 1.000 | 1.000 | 1.000 | 1.000 | 0.397 | 1.000 | - |


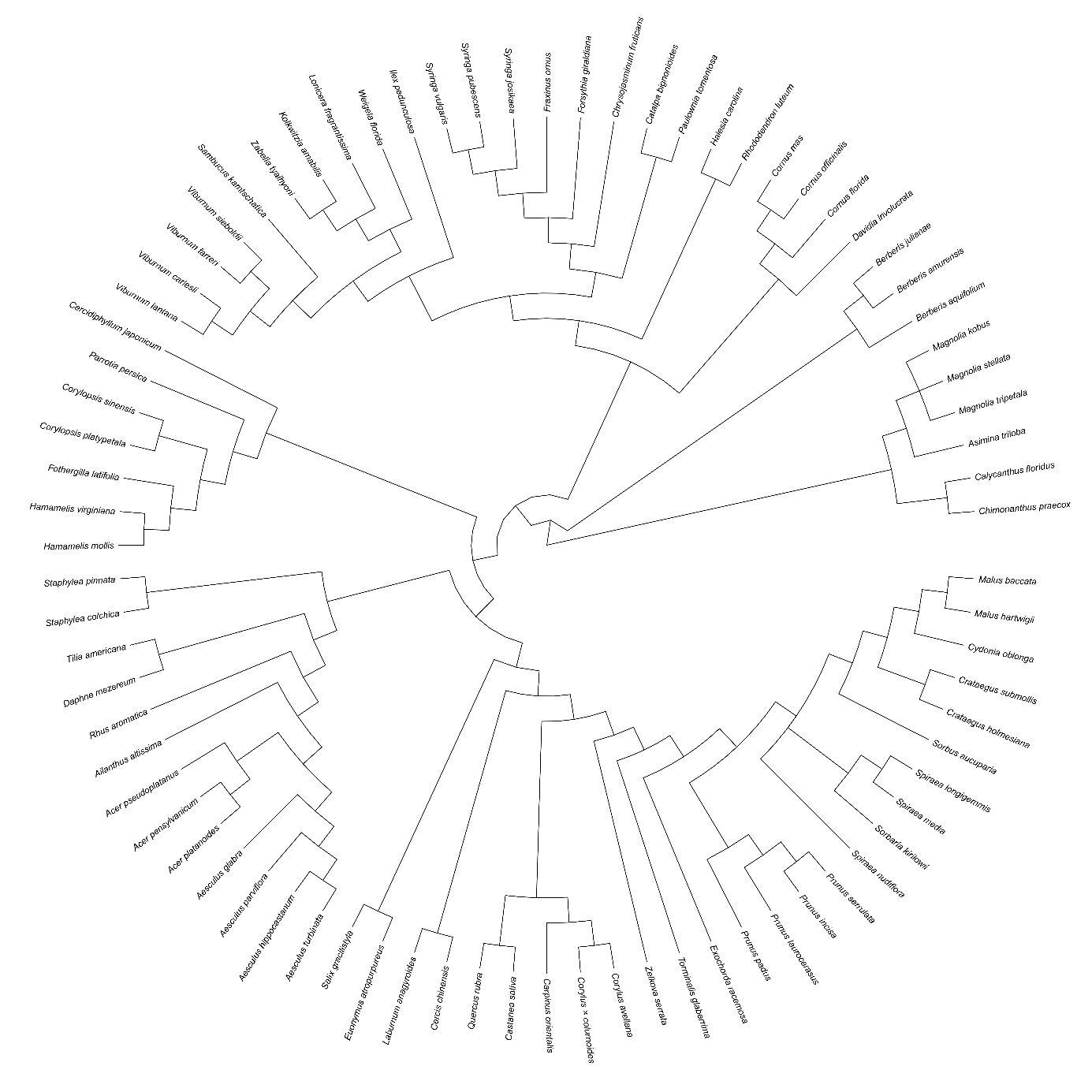


**Fig. S1.** Phylogenetic tree visualizing the evolutionary history of analyzed species.
